# p53-mediated regulation of electron transport chain and nucleotide synthesis during Newcastle disease virus infection

**DOI:** 10.1128/jvi.01576-25

**Published:** 2025-10-31

**Authors:** Changrun Zhao, Ning Tang, Jing Wang, Yang Qu, Lei Tan, Cuiping Song, Xusheng Qiu, Ying Liao, Tingrong Luo, Chan Ding, Yingjie Sun

**Affiliations:** 1 Laboratory of Veterinary Microbiology and Animal Infectious Diseases, College of Animal Sciences and Veterinary Medicine, Guangxi University 12664, Nanning, Guangxi, China; 2 Department of Avian Infectious Diseases, Shanghai Veterinary Research Institute, Chinese Academy of Agricultural Science 118161 , Shanghai, China; 3 State Key Laboratory for Conservation and Utilization of Subtropical Agro-Bioresources, Guangxi University 12664, Nanning, Guangxi, China; 4 Jiangsu Co-innovation Center for Prevention and Control of Important Animal Infectious Diseases and Zoonoses, College of Veterinary Medicine, Yangzhou University 38043, Yangzhou, Jiangsu, China; 5 School of Agriculture and Biology, Shanghai Jiao Tong University 12474, Shanghai, Shanghai, China; University of Kentucky College of Medicine, Lexington, Kentucky, USA

**Keywords:** NDV, mitochondrial metabolism, electron transport chain, p53, reactive oxygen species, nucleotide synthesis

## Abstract

Mitochondria and their electron transport chain (ETC) constitute the central machinery for cellular energy metabolism and biosynthetic regulation. Disruption of the ETC leads to reactive oxygen species (ROS) production and metabolic imbalance, but its precise role in viral replication and infection remains to be elucidated. In this study, we used Newcastle disease virus (NDV), an important avian pathogen and a promising oncolytic virus, as a model to explore its relationship with cellular mitochondrial metabolism. We demonstrate that NDV infection induces varying degrees of mitochondrial fragmentation, membrane potential dissipation, and ROS production, especially in p53-null H1299 cells compared to p53-wild-type A549 cells. ETC impairment restricts NDV replication primarily by limiting aspartate and pyrimidine nucleotide biosynthesis, rather than through ROS-mediated cytotoxicity or energy depletion. Notably, NDV replication in p53-null cells is highly sensitive to ETC complexes I and III inhibition, which can be rescued by exogenous aspartate or uridine supplementation. Mechanistically, p53 serves as a metabolic buffer, protecting mitochondrial function and maintaining precursor availability during viral infection. These findings elucidate the selective and differential utilization of mitochondrial ETC components by NDV and reveal that p53 status shapes cellular susceptibility to NDV-induced metabolic stress. Our work highlights mitochondrial metabolism and p53 as potential targets for antiviral and oncolytic strategies against NDV.

**IMPORTANCE:**

This study uncovers the intricate relationship between Newcastle disease virus (NDV) infection and host cell mitochondrial metabolism, with a particular emphasis on the pivotal regulatory role of p53. As both an important avian pathogen and a promising oncolytic virus, NDV disrupts mitochondrial function and the electron transport chain, leading to p53-mediated alterations in cellular energy metabolism and redox homeostasis. Our findings not only deepen the understanding of NDV-mitochondria interactions but also highlight the central role of p53 in viral infection and oncolytic mechanisms. These insights provide a theoretical foundation and novel therapeutic targets for antiviral and anticancer strategies based on p53 or mitochondrial pathways.

## INTRODUCTION

Mitochondria serve as the metabolic hub of eukaryotic cells, maintaining cellular homeostasis through dynamic fusion-fission equilibrium (1). The cristae structure of the inner membrane forms the critical foundation for electron transport chain (ETC) function, where respiratory complexes I–IV assemble into supercomplexes to drive ATP production via oxidative phosphorylation (OXPHOS) (2). When mitochondrial damage occurs (such as excessive fission), it triggers aberrant cleavage of the cristae-organizing protein Opa1, leading to cristae swelling and disintegration (3). This structural disruption directly impairs ETC supercomplex assembly, manifesting as decreased electron transfer efficiency, increased electron leakage, and a marked elevation in reactive oxygen species (ROS) production above baseline levels, along with reduced OXPHOS capacity (4). Notably, ETC dysfunction further compromises nucleotide biosynthesis, creating shortages of essential metabolic precursors (5). Concurrently, the loss of inner membrane integrity causes abnormal depletion of key metabolites including NADH, exacerbating the cellular energy crisis. Importantly, this damage initiates a vicious cycle: excessive ROS not only directly damages mitochondrial DNA and membrane phospholipids but also oxidatively modifies respiratory chain proteins, perpetuating and amplifying ETC dysfunction (6, 7). Ultimately, this cascade leads to systemic dysregulation of the cellular metabolic network.

As the integrative converter of mitochondrial bioenergetics, metabolism, and signaling, the ETC constitutes a fate-deciding cellular apparatus whose functional state governs survival-death decisions. Mitochondrial complex I, the primary initiator of the ETC and the largest protein complex in this system, catalyzes the oxidation of NADH to NAD^+^ (NADH → NAD^+^ + H^+^ + 2e⁻). This NAD^+^ regeneration process fundamentally determines the metabolic flux through the TCA cycle (8). Consequently, complex I dysfunction results in NADH accumulation, which potently stagnates the TCA cycle and consequently blocks aspartate synthesis (5). Instead, this may shift toward driving aspartate production via pyruvate carboxylation and reductive carboxylation of glutamine as a compensatory pathway to make up for aspartate deficiency (9). During electron transfer to ubiquinone (UQ → UQH₂), UQ accepts two electrons from the dihydroorotate-to-orotate conversion catalyzed by dihydroorotate dehydrogenase (DHODH), and UQ ultimately transfers the electrons to complex III. Thus, DHODH functions as a critical metabolic node that directly couples nucleotide metabolism with mitochondrial energy metabolism. Therefore, targeting DHODH has become a key target for many antiviral drugs (10, 11).

The ETC plays a complex yet pivotal role in viral infection, influencing viral pathogenesis through dual mechanisms of metabolic reprogramming and ROS-mediated regulation. Studies demonstrate that HIV reprograms ETC-dependent OXPHOS and glutaminolytic pathways to markedly enhance CD4^+^ T cell susceptibility to viral infection (12). Hepatitis C virus (HCV) infection also promotes moderate levels of ROS production to activate NF-κB, thereby enhancing viral infectivity (13). Such physiological ROS concentrations facilitate viral replication through two concerted mechanisms: suppressing mitochondrial antiviral-signaling protein (MAVS)-mediated interferon production (14) while simultaneously activating the Nrf2/glutathione (GSH) antioxidant system to delay host cell apoptosis (15). Conversely, excessive ROS accumulation resulting from ETC dysfunction exerts potent antiviral effects by promoting viral RNA degradation and enhancing innate immune responses, as observed in dengue virus infection (16). These findings establish the ETC as a central regulator of virus-host interactions. Notably, while the ETC’s role in regulating nucleotide metabolism through DHODH is well-established (17, 18), its specific impact on viral nucleotide synthesis remains largely unexplored.

Newcastle disease, caused by the Newcastle disease virus (NDV), is characterized by acute respiratory symptoms, neurological dysfunction, and high lethality, posing a significant threat to the global poultry industry (19). In addition, NDV has emerged as a promising oncolytic virus due to its selective and efficient replication in tumor cells. Recent advances in viral engineering have led to the development of recombinant NDV strains capable of safely lysing tumors in both murine models and human clinical trials, underscoring its therapeutic potential (17, 18). Our previous studies demonstrated that NDV exhibits robust replication in tumor cells, highlighting its ability to efficiently exploit host metabolic resources to facilitate viral propagation (20–22). Subsequent investigations revealed that NDV actively reprograms mitochondrial function through multiple mechanisms: restructuring OXPHOS (23), diverting glutamine catabolism (24), and dysregulating free calcium (Ca²^+^) signaling (25). In this study, we investigated the selective dependence of NDV replication on the ETC across different tumor cell types. Our findings reveal that NDV induces varying degrees of mitochondrial dysfunction and ROS production in tumor cells depending on their p53 status. As a critical tumor suppressor, p53 mediates mitochondrial fission to maintain cellular quality control by both eliminating damaged organelles and preserving bioenergetic metabolism through TCA cycle and OXPHOS protection (20–22). Notably, NDV replication demonstrates heightened sensitivity to ETC inhibitors in p53-null cells compared to p53-WT cells. Mechanistic studies further established that viral replication differentially relies on complex I-mediated regulation of aspartate biosynthesis and complex III-dependent maintenance of pyrimidine nucleotide synthesis. These results collectively demonstrate that p53 plays a protective role in mitigating NDV-induced ETC damage while maintaining cellular homeostasis. Our work, therefore, provides definitive evidence for the selective and differential utilization of mitochondrial ETC components during NDV replication, highlighting potential therapeutic targets for oncolytic virotherapy.

## MATERIALS AND METHODS

### Cells and viruses

A549, NCI-H1299, and BHK-21 cells were purchased from the Cell Bank of the Shanghai Institute of Biochemistry and Cell Biology, Chinese Academy of Science (http://www.cellbank.org.cn). A549 and NCI-H1299 cells were cultured in RPMI-1640 medium supplemented with 10% fetal bovine serum (FBS) (FSP500, ExCell Bio), BHK-21 cells were cultured in Dulbecco’s Modified Eagle Medium (DMEM) supplemented with 10% FBS under standard culture conditions (37°C, 5% CO₂, and 95% air) in a humidified incubator. NDV Herts/33 strain (a virulent, velogenic strain; GenBank accession No. AY741404.1) was provided by the China Institute of Veterinary Drug Control (Beijing, China).

### Antibodies and reagents

Mouse monoclonal anti-NP antibody was prepared in our laboratory. Mouse monoclonal anti-β-actin (66009-1-Ig) and rabbit polyclonal anti-p53 (10442-1-AP) were purchased from Proteintech. Rabbit monoclonal anti-HA-Tag (3724) antibody was purchased from Cell Signaling Technology. The Crystal Violet Staining Solution (C0121), Mito-Tracker Red CMXRos (C1035), and the JC-1 mitochondrial membrane potential assay kit (C2006), commonly used to detect mitochondrial membrane potential (ΔΨm), were purchased from Beyotime. Rotenone (HY-B1756), carbonyl cyanide 4-(trifluoromethoxy) phenylhydrazone (FCCP, HY-100410), and oligomycin (HY-N6782) were purchased from MedChemExpress. Antimycin A (GC49360) was purchased from GlpBio.

### Transfection of plasmids or siRNAs

For *TP53* knockdown, we used a validated siRNA sequence (siTP53) as previously described in reference (12). siRNA sequences were synthesized from Genpharma. The HA-tagged p53 expression plasmid (HA-p53) was constructed by cloning the full-length coding sequence of human *TP53* (RefSeq accession: NM_000546) into the pCMV-HA vector (Clontech Laboratories). siTP53 or HA-p53 plasmid was transfected using Lipofectamine 2000 (Thermo Fisher Scientific) according to the manufacturer’s protocol. Briefly, cells were seeded in 6-well plates and cultured until reaching 60%–80% confluence (typically within 24 h). Transfection was performed using the dual-tube (A/B) lipofection method as previously optimized (20). Following transfection, cells were maintained for 24 (plasmid transfection) or 48 h (siRNA transfection) to allow for sufficient transgene expression before subsequent analysis.

### Confocal microscopic analysis of mitochondrial morphology

Mitochondrial morphology was analyzed using Mito-Tracker Red CMXRos. A549 or H1299 cells grown to 80% confluence in 6-well plates were mock infected or infected with NDV for 6 and 18 h. Cells were loaded with 100 nM Mito-Tracker Red CMXRos in culture medium for 30 min at 37°C under 5% CO₂. After removing residual probe through three phosphate buffered saline (PBS) washes, fresh pre-warmed medium was added for live-cell imaging. Mitochondrial images were acquired using a confocal laser scanning microscope (LSM 880, Zeiss) with 579/599 nm excitation/emission settings. Fluorescence quantification and morphological analysis were performed using ImageJ with threshold-based masking.

### ΔΨm assay

Cells were seeded uniformly in 6-well plates and allowed to adhere overnight. Following experimental treatment protocols, cellular responses were assessed using laser scanning confocal microscopy. Briefly: JC-1 stock solution (200×) was diluted in ultrapure water at 50 µL: 8 mL to prepare the working solution. After removing the culture medium, cells were gently washed with PBS. Subsequently, 1 mL of JC-1 working solution was added to each well, followed by incubation at 37°C for 20 min in a humidified atmosphere. Following incubation, cells were washed twice with 1 × JC-1 staining buffer and maintained in 2 mL of fresh culture medium for immediate confocal microscopy observation.

### Mitochondrial respiration analysis

Cellular oxygen consumption rate (OCR) was quantified using the Agilent Seahorse XFe96 Analyzer (Seahorse Bioscience). For the assay, cells were seeded at a density of 20,000 cells per well in XFe96 microtiter plates and allowed to adhere overnight. Prior to measurement, the culture medium was replaced with 175 µL of pre-warmed assay medium, and plates were incubated for 1 h without CO_2_ to ensure proper pH equilibration. During the OCR assessment, pharmacological agents were sequentially administered to evaluate mitochondrial function: first, oligomycin (2 µM) was injected to inhibit ATP synthase, followed by the mitochondrial uncoupler FCCP (1 µM) to induce maximal respiration. Finally, a combination of respiratory chain inhibitors—Antimycin A (0.5 µM) and rotenone (0.5 µM)—was introduced to completely inhibit electron transport. This sequential injection protocol facilitates the comprehensive assessment of critical mitochondrial functional parameters, including basal OCR, maximal respiratory capacity, spare respiratory capacity, and ATP-linked respiration.

### Detection of intracellular ROS by flow cytometry

ROS levels were measured using a ROS assay kit (Beyotime, S0033). A549 or H1299 cells were cultured in 6-well plates and subjected to various treatments including NDV infection or pretreatment with different inhibitors for specified durations. Rotenone pretreatment (30 min) served as a positive control. Cell samples were processed using the following protocol: after removing the culture medium, 1.5  mL of diluted dichloro-dihydro-fluorescein diacetate (DCFH-DA; 10  µM in serum-free medium), a cell-permeable fluorescent probe for detecting intracellular ROS (Ex/Em  =  488/525  nm), was added to each well. Cells were incubated with the probe for 20 min at 37°C in a humidified incubator. Following incubation, cells were washed twice with PBS and subsequently detached using trypsin digestion. The cell suspension was centrifuged and resuspended in 300 µL PBS for final analysis. Flow cytometry analysis was performed using 488 nm excitation and 525 nm emission wavelengths.

### Extracellular viral titer assay

To determine the viral titer in cell culture supernatants, a classical plaque assay was performed (26). Briefly, BHK-21 cells were seeded uniformly in 24-well plates and incubated overnight. The supernatant was serially diluted (10^−1^ to 10^−6^) in serum-free DMEM. The BHK-21 cells were washed with PBS to remove residual serum, and 200 µL of the diluted supernatant was added to each well. After 2 h of incubation to allow viral adsorption, unbound viral particles were removed, and the cells were overlaid with 2% carboxymethyl cellulose. Following a 72 h incubation, the cells were fixed with 4% formaldehyde for 20 min and stained with 0.1% crystal violet for 20 min. Excess crystal violet was washed away with water. Plaques were counted, and the viral titer was calculated as plaque-forming units per milliliter (PFU/mL).

### Western blot analysis

Cell samples were harvested from 6-well plates. After PBS washing and lysis with radioimmunoprecipitation assay lysis buffer (P0013C, Beyotime), the supernatant obtained through centrifugation was mixed with 5 × SDS-PAGE loading buffer, boiled for 15 min and then stored at −20°C. Protein separation was performed using 10% SDS-PAGE, followed by electrophoretic transfer onto nitrocellulose membranes (NC-a101-b105, Whatman). The membranes were blocked with 5% skimmed milk in Tris-buffered saline containing 0.05% Tween 20 for 1 h at room temperature and then incubated with primary antibodies overnight at 4°C. After thorough washing, membranes were incubated with secondary antibodies for 2 h at room temperature. Following additional washes, antibody-antigen complexes were visualized using a LumiQ horseradish peroxidase (HRP) detection kit (Share-bio Biotechnology) and imaged with a multi-chemiluminescence detection system (Tanon 5200, Tanon).

### Statistical analysis

Data represent the means of at least three independent experiments. Statistical differences were ascertained using GraphPad Prism 8.0 software. Data were expressed as means ± standard deviations. Significance was determined with the two-tailed independent Student’s *t* test (*P* < 0.05) between two groups. One-way analysis of variance followed by Tukey’s test was used to compare multiple groups (>2).

## RESULTS

### NDV induces varying degrees of mitochondrial fragmentation and ROS production in different tumor cells

**Fig 1:**
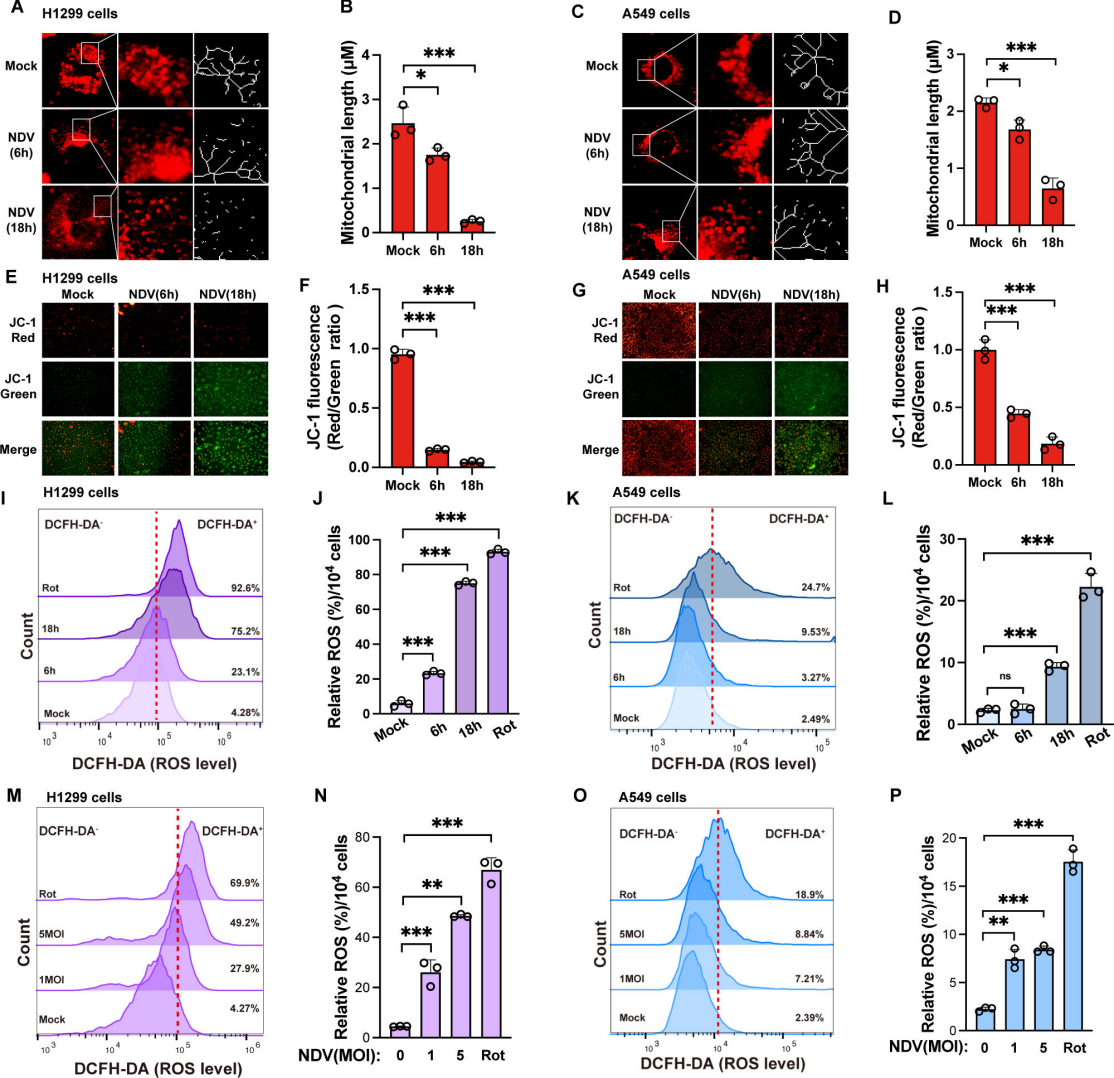
NDV induces mitochondrial fragmentation and ROS production in tumor cells (**A–D**) H1299 (**A**) and A549 (**C**) cells were infected with NDV at an MOI of 1 for 6 and 18 hpi. Cells were stained live with MitoTracker Red. Mitochondrial morphology was captured using confocal microscopy (red: mitochondria). Mitochondrial length was measured using ImageJ software (**B and D**). (**E–H**) H1299 (**E**) and A549 (**G**) cells were infected with NDV at an MOI of 1 for 6 hpi and 18 hpi. Cells were assessed for ΔΨm using JC-1 staining, with results analyzed by fluorescence microscopy and quantified (**F and H**). (**I–L**) H1299 (**I and J**) and A549 (**K and L**) cells were infected with NDV at an MOI of 1 for 6 hpi and 18 hpi. Cells were assessed by analyzing the fluorescence intensity of the DCFH-DA probe using FlowJo software, and rotenone was used as a positive control. (**M–P**) H1299 (**M and N**) and A549 (**O and P**) cells were infected with NDV at an MOI of 1 or 5 for 18 hpi. Cells were assessed by analyzing the fluorescence intensity of the DCFH-DA probe using FlowJo software, and rotenone was used as a positive control. Data are presented as means from three independent experiments. ns, no significance, *P < 0.05, **P < 0.01, ***P < 0.001.

Mitochondria, cellular powerhouses, are regulated by viral infections, affecting energy and metabolism. Their fusion and fission states reflect activity levels to meet energy needs (14, 15). To examine mitochondrial structural changes during NDV infection, we used MitoTracker probes for time-course analysis in various tumor cell lines. The results showed that NDV infection induced a gradual shift from elongated to fragmented mitochondrial morphology, with H1299 cells exhibiting more pronounced changes than A549 cells under equivalent infection conditions (Fig. 1A through D). Mitochondrial fragmentation, often linked to a loss of ΔΨm, was evaluated using JC-1 staining, a sensitive indicator of ΔΨm. Both A549 and H1299 cells displayed significant ΔΨm dissipation following NDV infection, but A549 cells exhibited greater resistance to ΔΨm loss compared to H1299 cells (Fig. 1E through H). Mitochondrial fragmentation, coupled with reduced ΔΨm, impairs ETC function and results in elevated ROS emission. To assess whether NDV infection induces intracellular ROS production, we measured ROS levels in A549 and H1299 cells at multiple time points post-infection. The results showed that ROS production increased by 21.6-fold in H1299 cells and by 9.9-fold in A549 cells after Rot treatment. Following NDV infection, ROS levels in H1299 cells increased by 5.4-fold at 6 h post-infection (hpi) and 17.6-fold at 18 hpi, compared to 1.3- and 3.8-fold increases in A549 cells (Fig. 1I through L). Consistent results were observed in NDV-infected cells at varying multiplicities of infection (MOIs) (Fig. 1M through P). DMSO, the solvent for rotenone, did not induce an increase in intracellular ROS levels (Fig. S1A and B). These findings indicate that, compared to A549 cells, H1299 cells are more sensitive to mitochondrial fragmentation and ROS production in response to NDV infection.

### Differential sensitivity of mitochondrial ETC to NDV infection in H1299 and A549 cells

**Fig 2:**
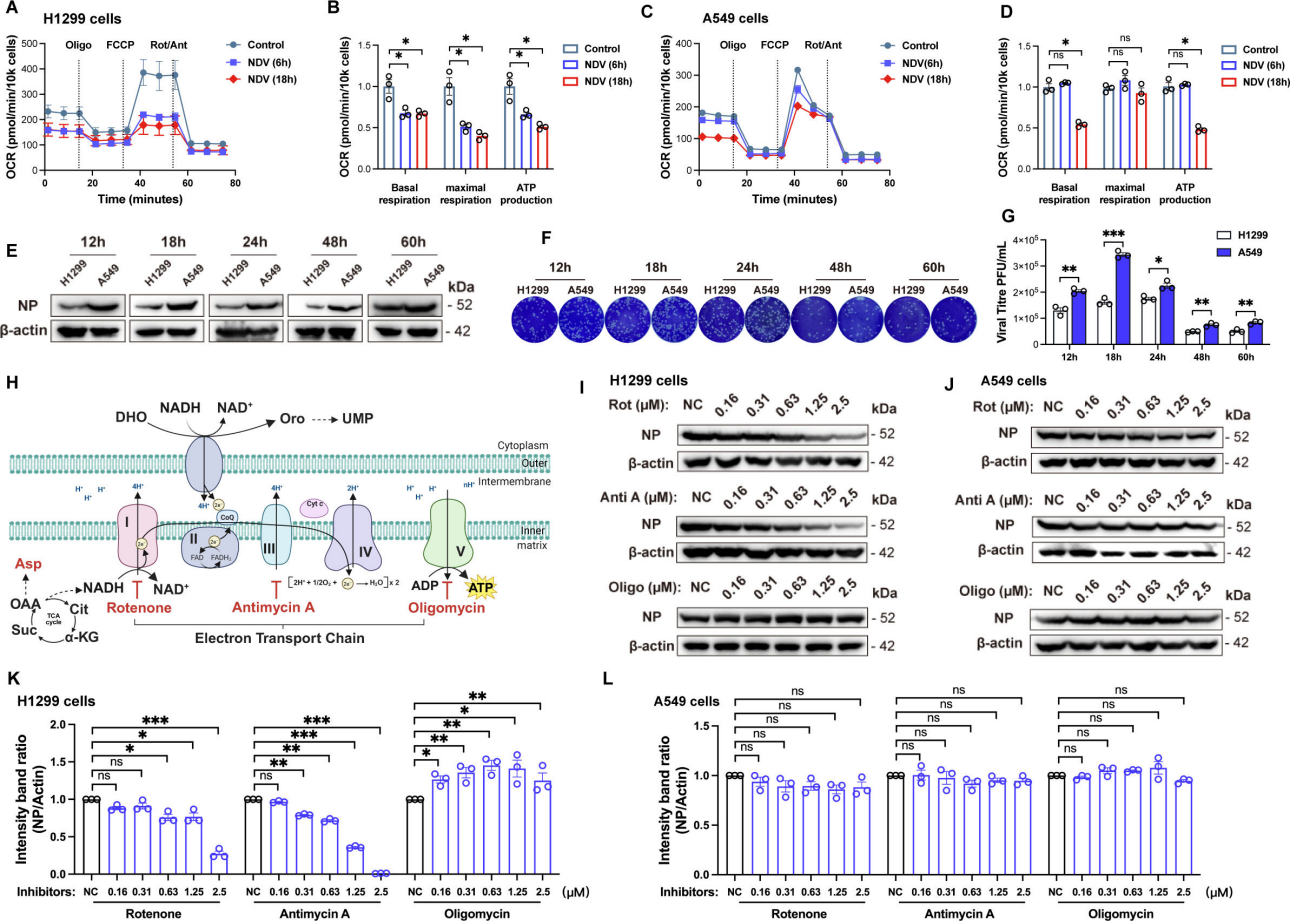
NDV infection differentially impairs mitochondrial ETC function in H1299 and A549 cells (**A–D**) H1299 (**A**) and A549 (**C**) cells were infected with NDV at an MOI of 1 for 6 hpi and 18 hpi. OCR was measured using the Agilent Seahorse XFe96 extracellular flux analyzer. Basal/maximal respiration and ATP production were analyzed using GraphPad software (**B and D**). (**E–G**) A549/H1299 cells were infected with NDV at an MOI of 1. Cells were harvested at 12, 18, 24, 48, and 60 hpi. Western blotting was employed to detect the protein levels of NDV-NP and β-actin (**E**). Cell supernatant was collected and the viral titer was determined by plaque assay (**F and G**). (**H**) Schematic representation of the synergistic effect of the ETC on aspartate and pyrimidine biosynthesis. Asp: aspartate; OAA: oxaloacetic acid; Suc: succinate; Cit: citrate; α-KG: α-ketoglutaric acid. DHO: dihydroorotate; Oro: orotate. The schematic figures were created with BioRender software (BioRender, Toronto, Ontario, Canada). (**I–L**) H1299 (**I**) and A549 (**J**) cells were infected with NDV at an MOI of 1 for 12 hpi and were treated with varying concentrations of ETC inhibitors (rotenone, antimycin A, and oligomycin) during this period. NC (negative control) represents cells treated with DMSO. Western blotting was employed to detect the protein levels of NDV-NP and β-actin. Grayscale quantification of NP/actin ratios from WB data of H1299 (**K**) and A549 cells (**L**). Data are presented as means from three independent experiments. ns, no significance,*P < 0.05, **P < 0.01, ***P < 0.001.

Since the mitochondrial ETC is the main source of cellular ROS and NDV infection induces mitochondrial fragmentation and elevates ROS levels, these findings collectively suggest a potential impairment of mitochondrial OXPHOS capacity. To quantify mitochondrial OXPHOS alterations following NDV infection, we measured the real-time OCR at various time points. The results showed that NDV infection significantly reduced OCR in H1299 cells as early as six hpi (Fig. 2A). In contrast, no significant decrease in OCR was observed in A549 cells at this stage (Fig. 2C). However, by the late stage of infection (18 hpi), both cell lines exhibited markedly reduced OCR levels, indicating progressive mitochondrial dysfunction with prolonged viral infection (Fig. 2A and C). Further analysis of basal respiration, maximal respiration, and ATP production revealed that H1299 cells exhibit a reduced capacity to respond and adapt to NDV infection compared to A549 cells (Fig. 2B and D). These findings suggest that NDV infection impairs the ETC in lung cancer cell lines, with H1299 cells being more sensitive to NDV-induced ETC damage than A549 cells. We questioned whether phenotypes such as mitochondrial fragmentation, increased ROS production, and ETC impairment were related to viral replication efficiency. To investigate this, we evaluated NDV replication in H1299 and A549 cells at the same MOI. If the severe mitochondrial dysfunction in H1299 cells were a consequence of deficient viral replication, lower replication would be expected to result in less mitochondrial impairment. However, despite H1299 cells exhibiting lower viral replication than A549 cells (Fig. 2E through G), they displayed more severe mitochondrial dysfunction. This inverse relationship suggests that mitochondrial dysfunction in these cells is not attributable to differences in viral replication efficiency. The different sensitivities to NDV-induced ETC damage in H1299 and A549 cells prompted us to examine the role of the ETC in viral replication. We used rotenone, antimycin A, and oligomycin to inhibit complexes I, III, and V, respectively. Mechanistically, rotenone blocks electron transfer from NADH to ubiquinone, and antimycin A prevents transfer from ubiquinol to cytochrome c; both halt ETC flow, increase upstream electron leakage, and promote ROS generation. Oligomycin blocks proton re-entry into the matrix, stopping ATP synthesis and inducing membrane hyperpolarization, which can indirectly elevate ROS (Fig. 2H). Rotenone and antimycin A markedly reduced NDV replication in H1299 cells, as evidenced by decreased NP protein expression (Fig. 2I and K), whereas A549 cells were unaffected (Fig. 2J and L). For rotenone, this inhibitory effect was apparent only at higher concentrations, likely because substantial complex I inhibition is required to significantly disrupt mitochondrial function and thereby interfere with NDV replication. In contrast, oligomycin had no appreciable effect on NDV NP expression in either cell line (Fig. 2I through L). DMSO, the solvent for rotenone, did not affect NDV replication (Fig. S1C). Together, these results demonstrate that NDV infection compromises mitochondrial function in lung cancer cells, with H1299 cells displaying greater vulnerability to ETC impairment, which in turn restricts viral replication.

### ETC dysfunction restricts NDV replication independently of ROS

**Fig 3:**
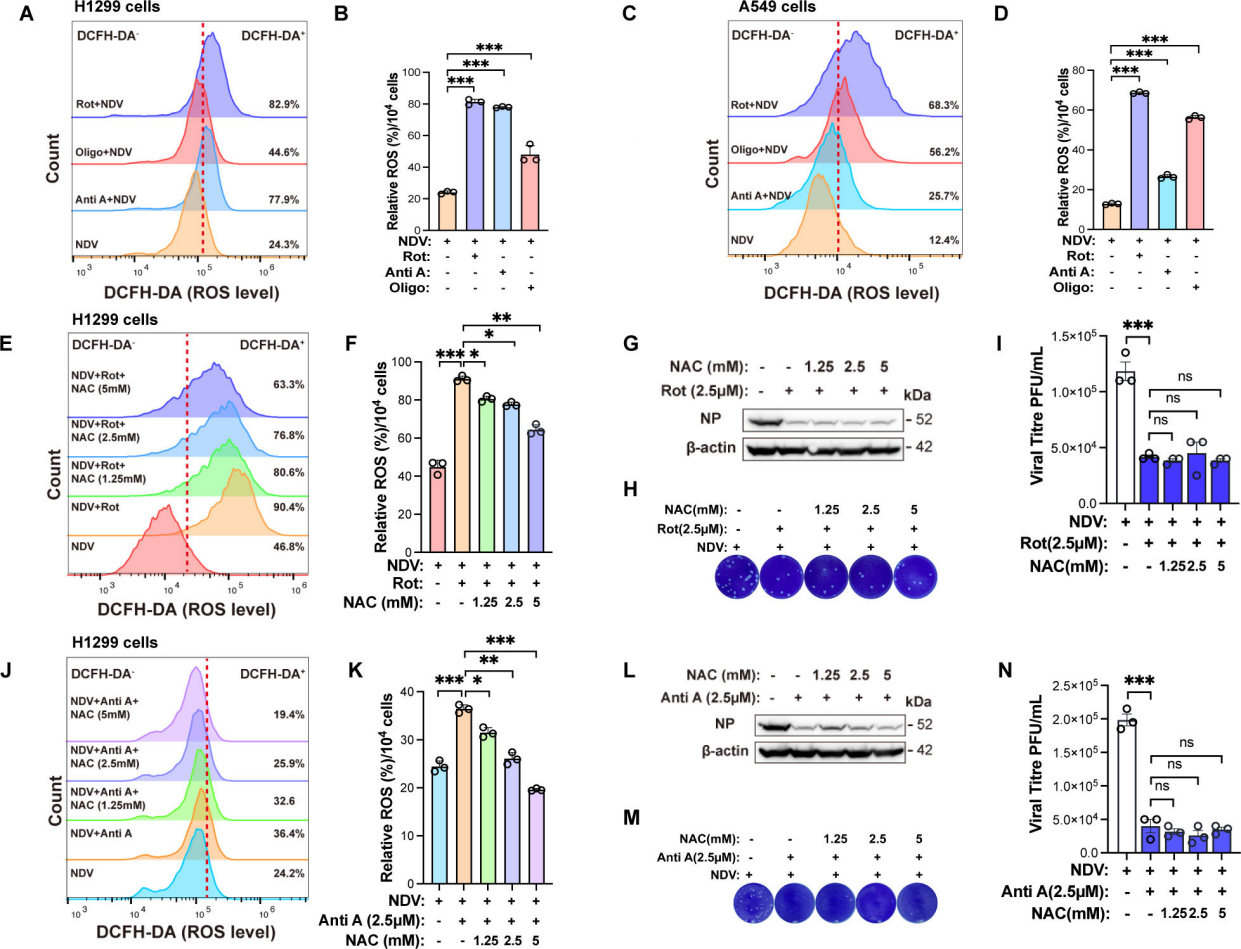
ETC dysfunction suppresses NDV replication through a ROS-independent mechanism (**A and B**). H1299 cells infected with NDV at an MOI of 1 for 12 hpi were treated with ETC inhibitors (rotenone, antimycin A, and oligomycin) or DMSO (solvent control) during this period. Intracellular ROS levels were quantified via flow cytometry using DCFH-DA staining (**A**), with fluorescence intensity analyzed by FlowJo software (**B**). (**C and D**) A549 cells infected with NDV at an MOI of 1 for 12 hpi were treated with ETC inhibitors (rotenone, antimycin A, and oligomycin) or DMSO (solvent control) during this period. Intracellular ROS levels were quantified via flow cytometry using DCFH-DA staining (**C**), with fluorescence intensity analyzed by FlowJo software (**D**). (**E and F**) H1299 cells infected with NDV at an MOI of 1 for 12 hpi were treated with rotenone and NAC at varying concentrations, with DMSO as the solvent control. Intracellular ROS levels were quantified via flow cytometry using DCFH-DA staining (**E**), with fluorescence intensity analyzed by FlowJo software (**F**). (**G**) Western blotting was employed to detect the protein levels of NDV-NP and β-actin. (**H and I**) Cell supernatant was collected and the viral titer was determined by plaque assay. (**J and K**) H1299 cells infected with NDV at an MOI of 1 for 12 hpi were treated with antimycin A and NAC at varying concentrations, with DMSO as the solvent control. Intracellular ROS levels were quantified via flow cytometry using DCFH-DA staining (**J**), with fluorescence intensity analyzed by FlowJo software (**K**). (**L**) Western blotting was employed to detect the protein levels of NDV-NP and β-actin. (**M and N**) Cell supernatant was collected and the viral titer was determined by plaque assay. Data are presented as means from three independent experiments. ns, no significance, *P < 0.05, **P < 0.01, ***P < 0.001.

Since ETC impairment inhibits NDV replication in H1299 cells, we sought to elucidate the underlying mechanism by which the ETC influences NDV replication. ETC inhibition leads to a surge in ROS, which can induce cytotoxicity and potentially affect viral replication. As expected, treatment with ETC inhibitors significantly increased ROS levels in NDV-infected H1299 (Fig. 3A and B) and A549 cells (Fig. 3C and D). To determine whether the cytotoxicity from rotenone- or antimycin A-induced ROS contributes to the inhibition of NDV replication, we utilized N-acetylcysteine (NAC), a well-known ROS scavenger, during ETC blockade. NAC supplementation effectively reduced rotenone-induced ROS production (Fig. 3E and F) but failed to rescue the suppression of NDV NP protein expression (Fig. 3G) or extracellular viral titers (Fig. 3H and I). Similarly, NAC reduced antimycin A-induced ROS accumulation (Fig. 3J and K) but did not restore NDV replication or viral release (Fig. 3L through N). Furthermore, cell viability and apoptosis assays showed that neither the ETC inhibitors alone nor combined with NAC caused significant cytotoxicity under our conditions (Fig. S2), indicating that their inhibition of NDV replication was not due to drug-induced cell death. Together, these results indicate that NDV replication suppression by rotenone and antimycin A, which target ETC Complexes I and III, occurs independently of ROS toxicity and is more likely due to disruption of essential metabolic processes required for efficient NDV replication.

### Mitochondrial complexes I and III cooperatively support NDV replication by driving pyrimidine biosynthesis

**Fig 4:**
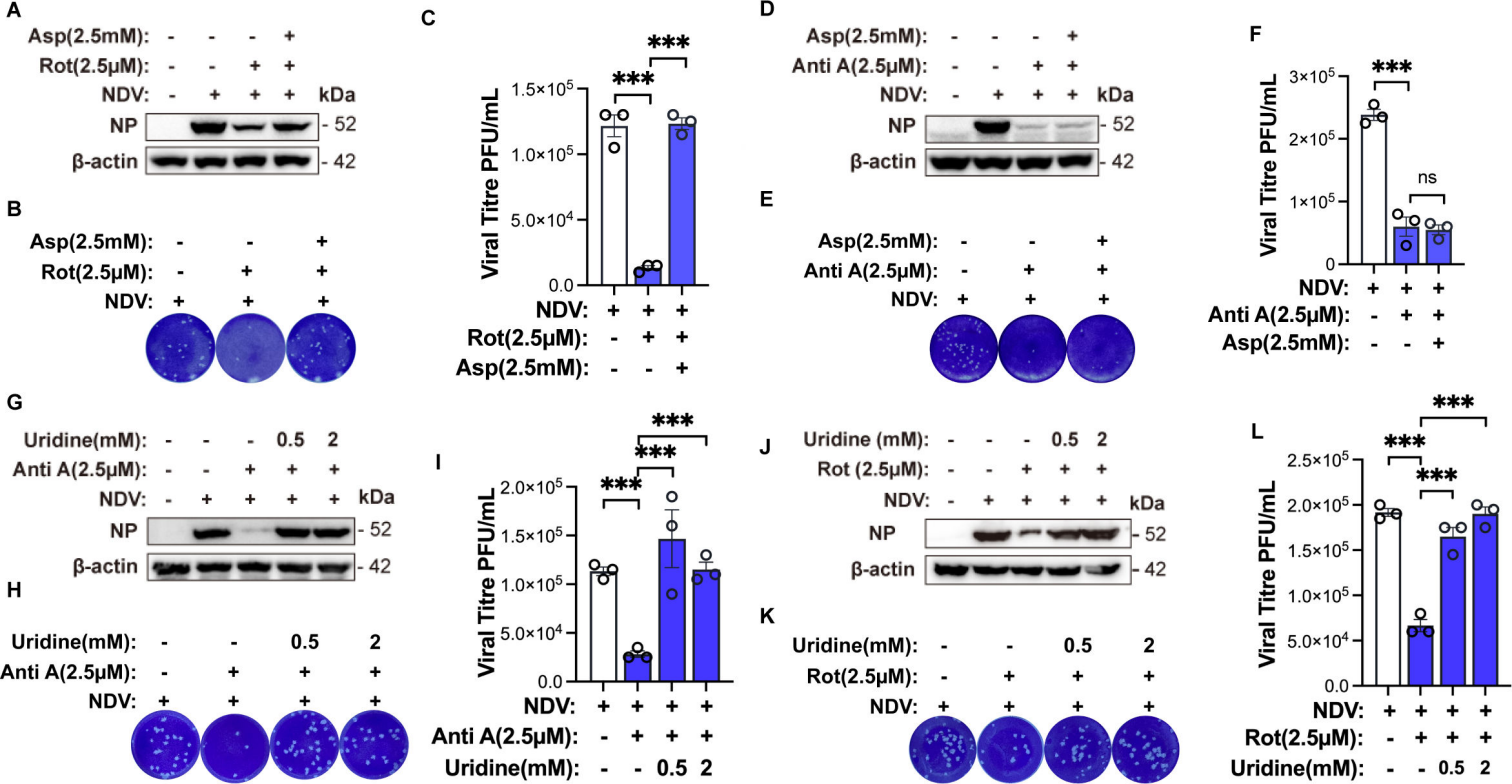
Mitochondrial complexes I and III sustain NDV replication via pyrimidine biosynthesis (**A–C**). H1299 cells infected with NDV at an MOI of 1 for 12 hpi were treated with rotenone and aspartate, with DMSO as the solvent control. Western blotting was employed to detect the protein levels of NDV-NP and β-actin (**A**). Cell supernatant was collected and the viral titer was determined by plaque assay (**B and C**). (**D–F**) H1299 cells infected with NDV at an MOI of 1 for 12 hpi were treated with antimycin A and aspartate, with DMSO as the solvent control. Western blotting was employed to detect the protein levels of NDV-NP and β-actin (**D**). Cell supernatant was collected and the viral titer was determined by plaque assay (**E and F**). (**G–I**) H1299 cells infected with NDV at an MOI of 1 for 12 hpi were treated with Antimycin A and uridine at varying concentrations, with DMSO as the solvent control. Western blotting was employed to detect the protein levels of NDV-NP and β-actin (**G**). Cell supernatant was collected and the viral titer was determined by plaque assay (**H and I**). (**J–L**) H1299 cells infected with NDV at an MOI of 1 for 12 hpi were treated with antimycin A and uridine at varying concentrations, with DMSO as the solvent control. Western blotting was employed to detect the protein levels of NDV-NP and β-actin (**J**). Cell supernatant was collected and the viral titer was determined by plaque assay (**K and L**). Data are presented as means from three independent experiments. ns, no significance, ***P < 0.001.

Since ROS is not responsible for the antiviral effect of complex I and III inhibition, we explored whether these complexes support NDV replication by supplying nucleotides. Complex I is essential for aspartate production through the TCA cycle (16, 23), while complex III works with DHODH to enable pyrimidine synthesis at the inner mitochondrial membrane (24, 25) (Fig. 2H). Both processes are important for nucleotide availability, which is necessary for viral replication. Therefore, we evaluated whether aspartate supplementation could rescue NDV replication in rotenone- and antimycin A-treated, NDV-infected H1299 cells. As shown in Fig. 4A through C, aspartate supplementation effectively restored both NDV NP protein expression and extracellular virus titers that were suppressed by rotenone, confirming that complex I supports viral replication primarily through aspartate synthesis. However, under the same conditions, aspartate resupply failed to restore NDV replication inhibited by antimycin A (Fig. 4D through F). Based on these observations, we speculated that antimycin A restricts NDV replication by inhibiting DHODH activity, thereby blocking pyrimidine synthesis. To test this hypothesis, we supplemented uridine to bypass the pyrimidine nucleotide limitation imposed by antimycin A-induced DHODH inhibition. The results showed that uridine supplementation markedly rescued both NDV NP protein expression and extracellular viral titers suppressed by antimycin A (Fig. 4G through I), confirming that NDV replication depends on the functional connection between mitochondrial complex III and DHODH for *de novo* pyrimidine biosynthesis. Furthermore, we propose that NDV utilizes complex I-derived aspartate to fuel pyrimidine synthesis, supporting its replication. Consistently, uridine supplementation also rescued NDV replication inhibited by rotenone (Fig. 4J through L). Meanwhile, supplementation with aspartate or uridine alone at the tested concentrations did not significantly enhance viral replication (Fig. S3), suggesting that endogenous levels are sufficient to support NDV replication and that exogenous supply is effective only under conditions of ETC impairment. Collectively, these findings demonstrate that NDV replication critically depends on both complexes I and III to support pyrimidine nucleotide biosynthesis.

### NDV stabilizes the ETC through p53 to extend its replication cycle

**Fig 5:**
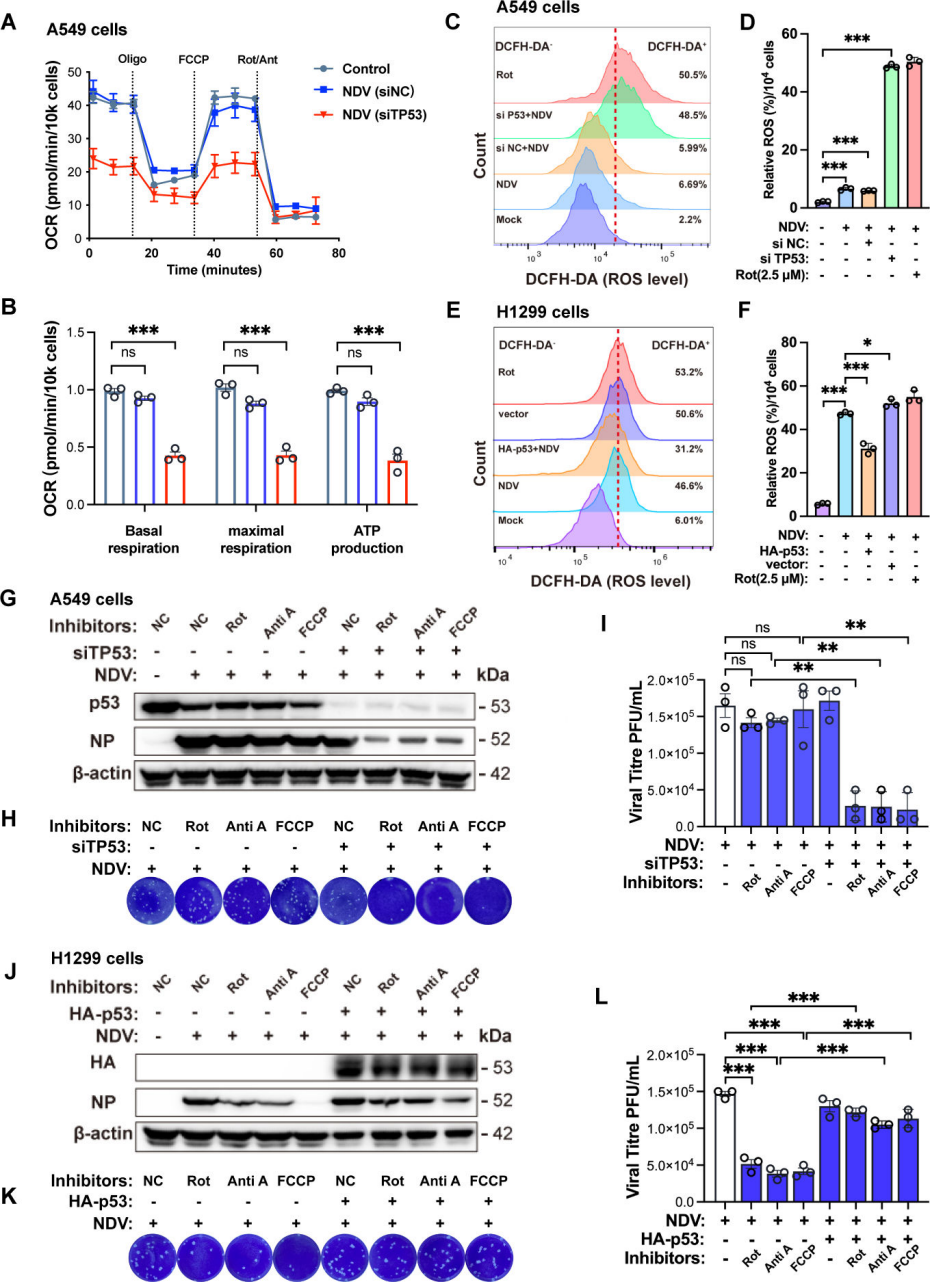
p53-mediated ETC stabilization promotes NDV replication (**A and B**) A549 cells were transfected with siRNA to knock down TP53. Twenty-four hours after transfection, cells were infected with NDV at an MOI of 1 for 6 hpi. OCR was measured using the Agilent Seahorse XFe96 extracellular flux analyzer (**A**). Basal/maximal respiration and ATP production were analyzed using GraphPad software (**B**). (**C and D**) A549 cells were transfected with siRNA to knock down TP53 and negative control siRNA (siNC). Twenty-four hours after transfection, cells were infected with NDV at an MOI of 1 for 18 hpi. Intracellular ROS levels were quantified via flow cytometry using DCFH-DA staining (**C**), with fluorescence intensity analyzed by FlowJo software (**D**). (**E and F**) H1299 cells were transfected with HA-p53. Twenty-four hours after transfection, cells were infected with NDV at an MOI of 1 for 18 hpi. Intracellular ROS levels were quantified via flow cytometry using DCFH-DA staining (**E**), with fluorescence intensity analyzed by FlowJo software (**F**). (**G–I**) A549 cells were transfected with siRNA to knock down TP53 and negative control siRNA (siNC). Twenty-four hours after transfection, cells were infected with NDV at an MOI of 1 for 12 hpi and were treated with varying concentrations of ETC inhibitors (rotenone, antimycin A, and FCCP) during this period. NC represents cells treated with DMSO. Western blotting was employed to detect the protein levels of NDV-NP, TP53, and β-actin (**G**). Cell supernatant was collected and the viral titer was determined by plaque assay (**H and I**). (**J–L**) H1299 cells were transfected with HA-p53. Twenty-four hours after transfection, cells were infected with NDV at an MOI of 1 for 12 hpi and were treated with varying concentrations of ETC inhibitors (rotenone, antimycin A, and FCCP) during this period. NC represents cells treated with DMSO. Western blotting was employed to detect the protein levels of NDV-NP, HA, and β-actin (**J**). Cell supernatant was collected and the viral titer was determined by plaque assay (**K and L**). Data are presented as means from three independent experiments. ns, no significance, *P < 0.05, **P < 0.01, ***P < 0.001.

NDV infection in A549 (p53-WT) and H1299 (p53-null) lung cancer cells shows marked differences in mitochondrial OXPHOS, ROS production, and sensitivity to ETC inhibitors. We hypothesized that p53 may help maintain ETC homeostasis, thereby reducing ROS and counteracting the antiviral effects of ETC inhibition. To explore this, we knocked down *TP53* in A549 cells and overexpressed p53 in H1299 cells. As shown in Fig. 5A and B, *TP53* knockdown in A549 cells reduced mitochondrial OXPHOS upon NDV infection. Moreover, *TP53* knockdown significantly increased intracellular ROS (Fig. 5C and D), while p53 overexpression in H1299 cells decreased NDV-induced ROS production (Fig. 5E and F), indicating that p53 modulates the ETC to limit ROS accumulation during infection. Next, we assessed whether p53 influences cellular sensitivity to ETC inhibitors during NDV infection. FCCP, another ETC inhibitor, was added to globally uncouple OXPHOS by dissipating the proton gradient, thereby causing a more profound disruption of mitochondrial function. ETC inhibitors had little effect on NDV replication in A549 cells but significantly suppressed NDV NP protein expression and viral titers in *TP53* knockdown cells (Fig. 5G through I). Conversely, ETC inhibitors reduced NDV replication in H1299 cells, but this inhibitory effect was reversed by p53 overexpression (Fig. 5J through L). Taken together, these results demonstrate that p53 plays a protective role in maintaining mitochondrial function and mitigating the impact of ETC inhibition on NDV replication.

## DISCUSSION

**Fig 6:**
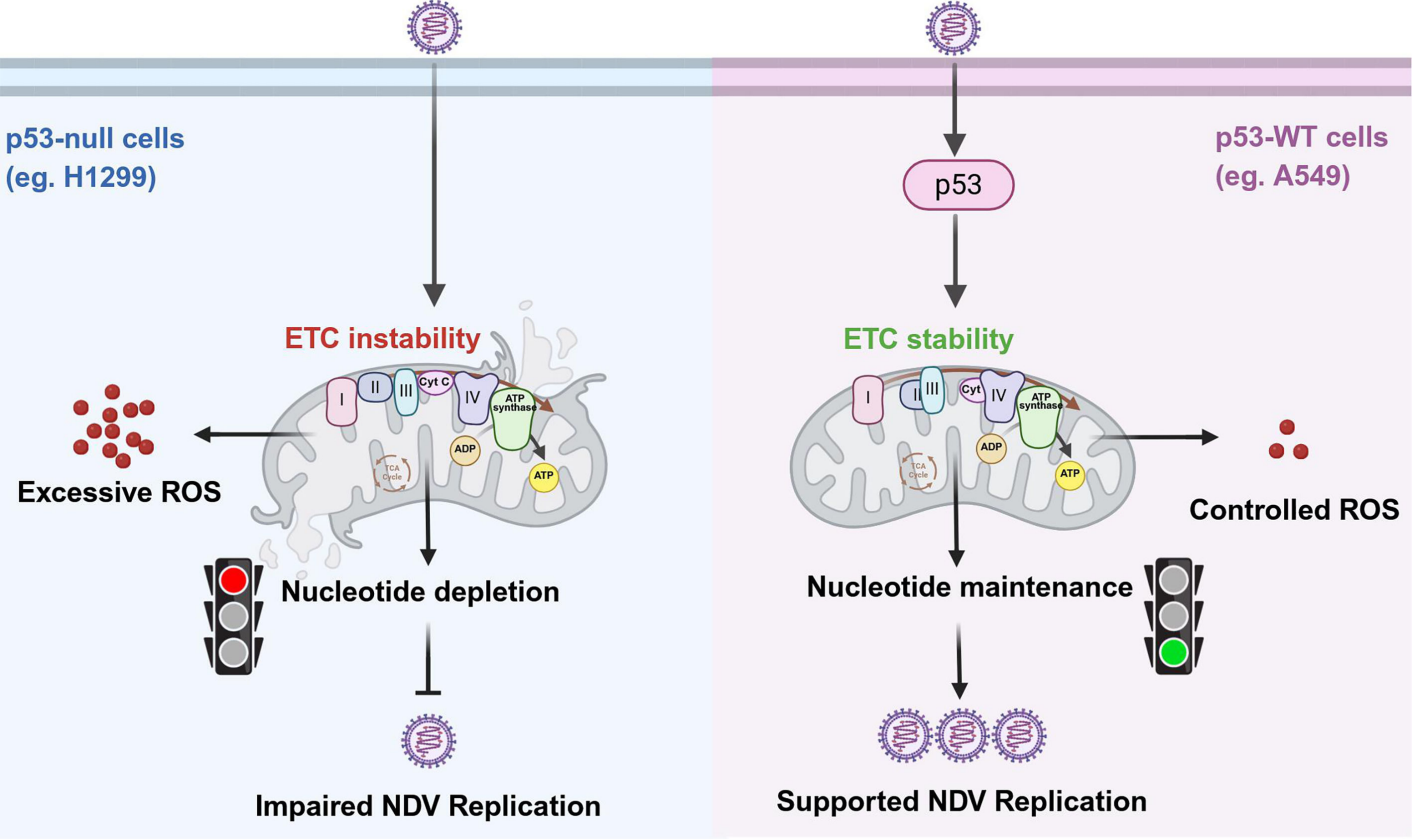
Model of NDV-induced p53 stabilization supporting ETC activity and nucleotide synthesis for viral replication. In the presence of p53, cells adapt to virus-induced metabolic stress and maintain nucleotide pools, supporting both cell survival and viral replication. Without p53, cells become reliant on intact ETC; ETC disruption quickly depletes nucleotides and impairs viral replication. Thus, while p53 preserves cellular stability, viruses may exploit this environment to enhance their own replication.

As the primary metabolic center of eukaryotic cells, mitochondria maintain cellular homeostasis through continual cycles of fusion and fission. While their primary role is ATP production via OXPHOS, their functions extend far beyond energy generation (27). The ETC, located in the mitochondrial inner membrane, is central to proton translocation, ATP synthesis, and involvement in various metabolic pathways (16, 28, 29). Previous studies, including ours, have shown that NDV disrupts mitochondrial fusion-fission balance, leading to OXPHOS restructuring, re-programming of glutamine catabolism, dysregulation of Ca²^+^ signaling, mitophagy, and cell death (22, 30, 31). In this study, we observed distinct phenotypes, such as differences in mitochondrial fragmentation, increased ROS production, and ETC impairment, between A549 and H1299 lung cancer cell lines following NDV infection. Investigating this divergence, we found that p53 acts as a “metabolic buffer,” preserving cellular homeostasis under various stresses. In the presence of p53, cells can endure virus-induced metabolic stress, rapidly adjust their metabolism, and maintain essential precursor supplies. Conversely, in the absence of p53, this protective mechanism is lost, making viral replication highly dependent on intact mitochondrial function; disruption of the ETC consequently leads to rapid depletion of nucleotide pools and impaired viral replication. Overall, p53’s fundamental role is to safeguard cellular stability, but viruses may exploit the stable environment maintained by p53 to facilitate their own replication (Fig. 6).

Mitochondria, serving as the central hubs for cellular energy production and metabolism, significantly influence viral replication when their homeostasis is disrupted. Numerous viruses manipulate mitochondrial function to alter the host cell’s metabolic environment, thereby promoting their own proliferation (20, 32, 33). It has been documented that infection by various viruses induces substantial accumulation of mitochondrial ROS (34, 35). Moderate ROS elevation can activate antiviral signaling pathways, modulate viral gene expression, and even facilitate viral genome replication (36); however, excessive ROS levels exert cytotoxic effects detrimental to both the virus and the host (37). Furthermore, many viruses upregulate glycolysis (the Warburg effect) to meet the increased demand for energy and biosynthetic precursors required for efficient viral replication. Our previous research demonstrated that NDV infection employs SIRT3 as a molecular switch governing cellular energy supply, downregulating mitochondrial OXPHOS while enhancing cytosolic glycolysis to generate energy (30). Notably, although NDV infection robustly induced both ROS accumulation and enhanced glycolytic flux in our studies, neither ROS scavenging (via NAC treatment) nor inhibition of mitochondrial ATP synthesis (via oligomycin treatment) significantly altered viral replication. These findings indicate that during NDV-host interactions, ROS levels and glycolytic activity are not primary limiting factors for viral replication. This contrasts with mechanisms reported for certain DNA viruses or lentiviruses (such as HIV)(38), suggesting that NDV, and potentially related paramyxoviruses, may depend more critically on alternative mitochondrial regulatory pathways for efficient replication.

Recent studies increasingly reveal that the mitochondrial ETC serves not only as the central hub for energy production but also participates in virus-host interactions by regulating the synthesis of diverse metabolites (39, 40). Our experimental results demonstrate that inhibitors targeting ETC complexes I and III (rotenone and antimycin A) significantly suppress NDV replication. This inhibitory effect aligns with previous reports for various other viruses, including SARS-CoV-2 and HIV-1 (41, 42), but those studies focused exclusively on ROS- and OXPHOS-mediated impacts on viral replication. Further validation revealed that these inhibitors primarily restrict viral replication by limiting the synthesis of aspartate and pyrimidine nucleotides, thereby disrupting the supply of key building blocks essential for viral nucleic acid and protein synthesis. Although ETC inhibitors induce elevated ROS levels, ROS scavenging failed to reverse their suppressive effect on viral replication. Consistent with the literature, this indicates that viral replication depends more critically on the biosynthetic functions of the mitochondrial ETC—specifically the supply of metabolites—than on energy production or ROS homeostasis (43). Our data further underscore the crucial role of mitochondrial metabolic reprogramming in virus-host interactions for supporting the viral life cycle.

During virus-host interactions, key host regulatory factors profoundly influence viral proliferative capacity. p53, classically recognized as the “guardian of the genome,” also orchestrates diverse functions encompassing metabolic homeostasis, antiviral responses, and cell fate determination (44, 45). Previous studies indicate that p53 can directly regulate ETC-associated proteins (e.g., SCO2), thereby promoting mitochondrial electron transport and OXPHOS to modulate ROS levels and metabolite synthesis (46). Our study demonstrates that NDV replication in p53-null H1299 cells exhibits enhanced sensitivity to ETC inhibitors, which can be reversed by exogenous aspartate and uridine supplementation. It was reported that p53 deficiency destabilizes the functional coupling between ETC complexes III and DHODH, a critical pyrimidine biosynthetic enzyme (29). This suggests that p53 supports viral replication by maintaining mitochondrial metabolic homeostasis to fulfill requisite biosynthetic demands. Concurrently, p53 can indirectly regulate viral replication in different infection contexts by influencing glycolysis, lipid metabolism, and even apoptotic pathways (47, 48). Furthermore, viruses have evolved diverse mechanisms to hijack or antagonize p53 function, facilitating their own replication and evasion of host immune surveillance. Consequently, p53 not only sustains ETC functionality but also potentially regulates the virus-host equilibrium through ancillary metabolic and signaling cascades.

Taken together, integrating our findings with existing literature reveals that mitochondrial dysregulation modulates viral replication through multiple mechanisms. However, in NDV-infected cells, impaired ETC function—through disruption of aspartate and pyrimidine biosynthesis—emerges as the key factor limiting viral replication. Furthermore, p53 further influences NDV replicative capacity by regulating the ETC and associated metabolic networks. These observations identify mitochondrial metabolism and functional p53 as important host determinants of NDV replication and oncolytic activity. While both processes are fundamental to cellular physiology and raise concerns about potential off-target effects, the differential dependency of tumor versus normal cells on these pathways may provide a therapeutic window. Thus, our work not only elucidates molecular mechanisms of virus-host interactions but also provides a mechanistic foundation for developing antiviral and oncolytic strategies that explicitly consider therapeutic selectivity and host cell-specific responses. Future studies warrant systematic investigation into the regulatory features of the p53-ETC-metabolism axis across diverse viruses, aiming to elucidate its broader significance in viral pathogenesis and host immune responses.

## Data Availability

All data supporting the results of this study are included in the article and its supplemental material and are also available from the corresponding author upon request, with no restrictions.

## SUPPLEMENTAL MATERIAL

Supplemental figures jvi.01576-25-s0001.pdf Figures S1 to S3.

ASM does not own the copyrights to Supplemental Material that may be linked to, or accessed through, an article. The authors have granted ASM a non-exclusive, world-wide license to publish the Supplemental Material files. Please contact the corresponding author directly for reuse.
