# Supplementary material for: p53-mediated regulation of electron transport chain and nucleotide synthesis during Newcastle disease virus infection
Source: J Virol. 2025 Oct 31;99(11):e01576-25. doi: 10.1128/jvi.01576-25 (PMC12645988; doi:10.1128/jvi.01576-25)
Supplement: Supplemental figures — Figures S1 to S3. [file jvi.01576-25-s0001.pdf]

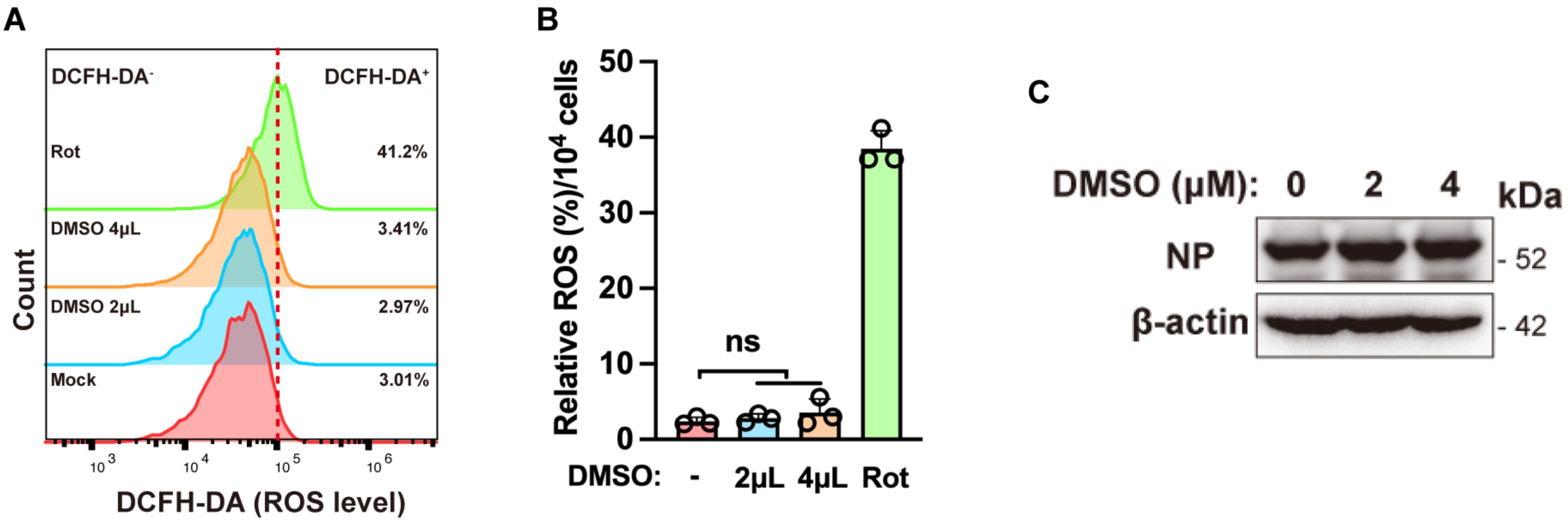

Supplemental Figure 1. Effect of DMSO on intracellular ROS levels and NDV replication. H1299 cells were treated with DMSO for 12 h; rotenone was used as a positive control (A and B). H1299 cells were infected with NDV at an MOI of 1 for 12 hpi and treated with DMSO. Western blotting was used to detect the protein levels of NDV-NP and β-actin (C).

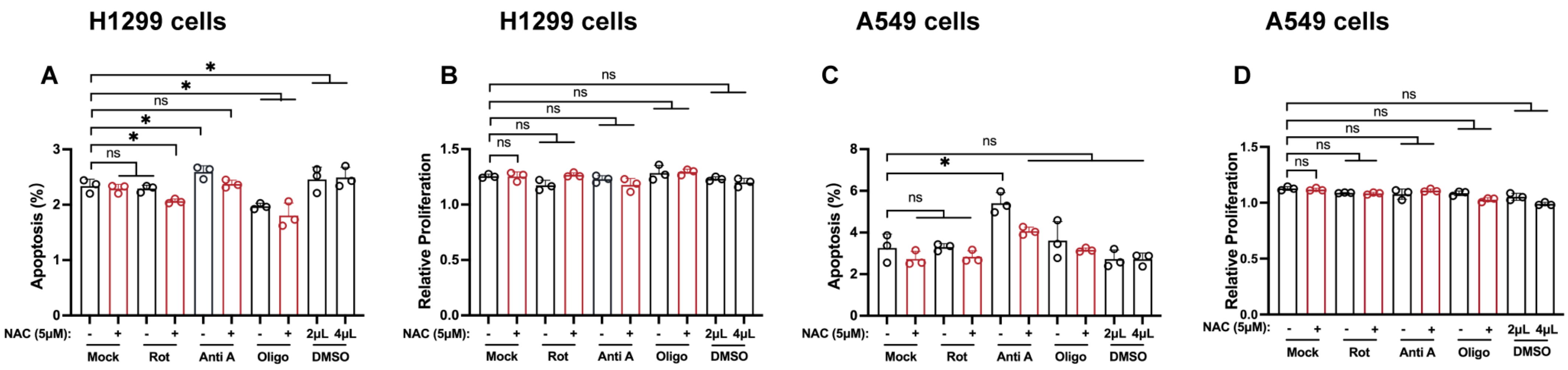

Supplemental Figure 2. Impact of ETC Inhibition and NAC on Cell Viability and Apoptosis. H1299 (A-B) and A549 (C-D) cells were treated with ETC inhibitors (Rotenone, Antimycin A, and Oligomycin, 2.5μM), DMSO, NAC for 12 h. Flow cytometry using the Cell Cycle and Apoptosis Analysis Kit (Beyotime, C1052) was used to detect the cell apoptosis (A and C). Cell proliferation rates were measured using Cell Counting Kit 8 (B and D).

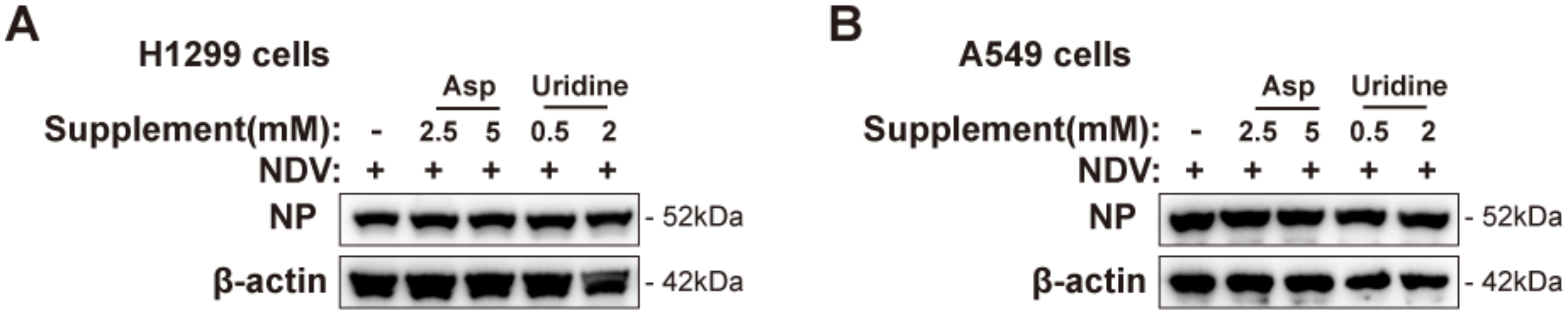

Supplemental Figure 3. Effect of Aspartate and Uridine Supplementation on NDV Replication. Aspartate and Uridine Effects on NDV. H1299 (A) and A549 (B) cells were infected with NDV at an MOI of 1 and, at 12 hpi, supplemented with varying concentrations of aspartate or uridine. NDV-NP and β-actin protein levels were detected by western blotting.
